# Vascular Aging and Atherosclerosis: The Modulatory Impact of Selenium—A Comprehensive Review

**DOI:** 10.3390/cells15110973

**Published:** 2026-05-25

**Authors:** Andrea Borghini, Mariangela Palazzo, Francesca Gorini

**Affiliations:** Institute of Clinical Physiology, National Research Council, 56124 Pisa, Italy; mariangelapalazzo@cnr.it

**Keywords:** selenium, selenoproteins, atherosclerosis, coronary heart disease, cardiovascular disease, cellular senescence, oxidative stress, inflammation, endothelial dysfunction, DNA damage

## Abstract

Selenium (Se), a vital trace element, plays a significant role in maintaining vascular health and may offer protective effects against atherosclerosis. Its actions are mediated through Se-dependent selenoenzymes and selenoproteins, which enhance antioxidant defense, modulate inflammatory responses, and promote autophagy. These processes collectively help prevent cellular senescence—a state associated with age-related vascular decline characterized by oxidative stress, DNA damage, pro-inflammatory activity, and endothelial dysfunction. Epidemiological evidence consistently shows that low Se status is associated with increased risk of atherosclerotic cardiovascular disease within a narrow concentration range. However, clinical trials have not demonstrated clear reductions in cardiovascular events or mortality with Se supplementation alone. Overall, current evidence indicates that Se modulates key mechanisms involved in vascular aging and atherosclerosis, particularly redox balance, immune activation, and vascular cell homeostasis. This comprehensive review summarizes current epidemiological, clinical, and experimental research on the role of Se in cardiovascular health. It underscores Se’s potential as a promising strategy for the prevention and treatment of atherosclerosis, while also acknowledging the complexities and nuances of its effects on vascular health. A deeper understanding of the cellular and molecular mechanisms involved could pave the way for targeted interventions aimed at reducing the burden of atherosclerotic cardiovascular disease.

## 1. Introduction

Atherosclerosis, a chronic and progressive inflammatory condition affecting the intima of medium- and large-sized arteries, characterized by endothelial dysfunction, lipid infiltration, and foam-cell formation, ultimately leading to plaque development, represents the leading cause of coronary heart disease (CHD), myocardial infarction (MI), ischemic stroke, and peripheral arterial disease [1,2,3]. Despite a global reduction in mortality from ischemic heart disease and ischemic stroke between 1990 and 2019 across all adult age groups—attributable to advances in public health strategies and early treatment—recent estimates indicate an increasing incidence of atherosclerosis over the same period [2]. This rise is largely driven by global population aging, lifestyle patterns characterized by high-fat dietary habits and insufficient physical activity, and a growing prevalence of chronic diseases such as hypertension and diabetes, which constitute major risk factors for atherosclerosis [2]. While atherosclerosis was historically viewed as a mere consequence of dyslipidaemia—driven by the accumulation of cholesterol-rich lipoproteins, primarily low-density lipoprotein (LDL), within the arterial intima—by the end of the last century, its inflammatory nature had been proposed, supported by the observation of circulating monocytes within the fatty streak [4,5]. Furthermore, accumulating evidence indicates that aging, defined as a progressive decline in cellular function caused by multiple factors, including genomic instability, telomere dysfunction, stem-cell exhaustion, and oxidative stress, contributes to the senescence of vascular and immune cells, thereby representing a pivotal driver in the promotion of atherosclerosis [3,6]. Indeed, although cellular senescence initially prevents the replication of damaged cells and thus protects against tumorigenesis, in the long term the secretion of inflammatory mediators and proteolytic enzymes by senescent cells—known as the senescence-associated secretory phenotype (SASP)—promotes vascular inflammation and remodelling [3,4].

Selenium (Se), an essential trace element involved in a wide range of physiological functions through its incorporation into selenoproteins as selenocysteine (SeCys), is recognized as one of the most potent antioxidant systems in humans [7,8,9]. As a component of selenoproteins, Se regulates the antioxidant defence system, primarily through glutathione peroxidase (GPx), thioredoxin reductase (TrxR), and selenoprotein P (SELENOP), attenuates inflammatory processes triggered by excess reactive species [10], enhances immune function [11], modulates thyroid activity [12], exerts anti-cancer effects [13], and supports male fertility [14] as well as female reproductive health [15]. An expanding body of research indicates that Se may also help reduce the risk of cardiovascular disease (CVD), primarily by mitigating oxidative-stress-induced damage, thereby reducing inflammation and endothelial dysfunction and inhibiting vascular cell apoptosis and vascular calcification [16,17,18]. Meanwhile, the antioxidant activity of Se, which reduces DNA damage and preserves telomere length, plays a crucial role in counteracting aging and preventing age-related diseases [19].

In this comprehensive literature review, we examine the current body of research from experimental and clinical studies on the role of Se in reducing the risk of atherosclerosis by acting at multiple levels, including the mitigation of cellular senescence, and discuss how Se supplementation may represent a valuable strategy to curb atherosclerosis and associated CVD. A bibliographic search was conducted in April 2026. The search was performed using PubMed, with keywords related to the topics mentioned above. To ensure comprehensive coverage, no restrictions were applied regarding publication type or study design. Only articles published in English were included, encompassing original research papers, reviews, and meta-analyses. The reference lists of the selected articles were also screened to identify additional relevant studies.

## 2. Atherosclerosis and Cellular Senescence

**Figure 1:**
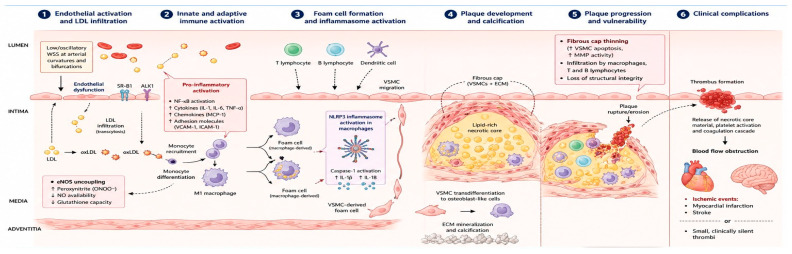
Key cellular and molecular mechanisms underlying the development and progression of atherosclerosis from endothelial dysfunction to plaque rupture and clinical complications. Disturbed blood flow patterns (low and oscillatory wall shear stress), particularly at arterial curvatures and bifurcations, promote endothelial dysfunction and increased permeability, facilitating the transcytosis and retention of circulating low-density lipoproteins (LDL) within the intimal layer. LDL undergoes oxidative modifications (oxLDL), triggering endothelial activation characterized by NF-κB signaling and the upregulation of pro-inflammatory cytokines (IL-1β, IL-6, TNF-α), chemokines (e.g., MCP-1), and adhesion molecules (VCAM-1, ICAM-1), which collectively promote leukocyte recruitment. Monocytes adhere to the activated endothelium, migrate into the intima, and differentiate into macrophages that internalize oxLDL through scavenger receptors, giving rise to lipid-laden foam cells. Concurrently, innate and adaptive immune responses involving macrophages, dendritic cells, and T and B lymphocytes amplify inflammation and plaque development. Macrophage activation contributes to NLRP3 inflammasome assembly, caspase-1 activation, and the release of IL-1β and IL-18, further sustaining vascular inflammation. As the lesion progresses, vascular smooth muscle cells (VSMCs) migrate from the media to the intima, proliferate, and contribute to extracellular matrix (ECM) production, leading to fibrous cap formation. Advanced plaques exhibit a lipid-rich necrotic core and may undergo calcification through VSMC transdifferentiation into osteoblast-like cells and ECM mineralization. Plaque progression is associated with structural instability due to fibrous cap thinning, increased matrix metalloproteinase (MMP) activity, VSMC apoptosis, and enhanced infiltration of inflammatory cells, resulting in plaque vulnerability. Ultimately, plaque rupture or erosion can expose thrombogenic material, promoting platelet activation and coagulation cascade initiation, thrombus formation, and blood flow obstruction, leading to ischemic clinical events such as myocardial infarction and stroke. Image generated with AI ChatGPT 5.0. Abbreviations: ALK1: activin A receptor-like type 1; ECM: extracellular matrix; eNOS: endothelial nitric oxide synthase; ICAM-1: intercellular adhesion molecular-1; IL: interleukin; LDL: low-density lipoprotein; MCP-1: monocyte chemoattractant protein-1, MMP: matrix metalloproteinase; NF-ĸB: nuclear factor kappa-light-chain-enhancer of activated B cells; NLRP3: NOD-like receptor protein 3; NO: nitric oxide; oxLDL: oxidized LDL; SR-B1: scavenger receptor B1; TNF-α: tumor necrosis factor alpha; VCAM-1: vascular cell adhesion molecule-1; VSMC: vascular smooth muscle cell; WSS: wall shear stress.

Atherosclerosis is an immunoinflammatory and fibroproliferative disease that originates in early childhood and progresses silently throughout adulthood [1], characterized by the progressive accumulation of lipids and fibrous tissue within the tunica intima of medium- and large-sized arteries [20,21]. The process begins with endothelial activation, which triggers a cascade of cellular and molecular events leading to atheroma formation and cardiovascular complications [20,21] (Figure 1). The vascular endothelium, a monolayer of endothelial cells (ECs), functions both as a selectively permeable barrier and as a sensor of mechanical and metabolic signals [20,21]. Turbulent blood flow at arterial curvatures and bifurcations—often exacerbated by hypercholesterolemia, hypertension, and type 2 diabetes—reduces wall shear stress, thereby promoting endothelial dysfunction and facilitating LDL infiltration into the tunica intima through transcytosis mediated by scavenger receptor B1 and activin A receptor-like type 1 [20,21]. Retained LDL undergo oxidative modification (oxLDL), triggering oxidative stress and activation of pro-inflammatory pathways such as nuclear factor kappa-light-chain-enhancer of activated B cells (NF-κB), which collectively promote cytokine release (interleukin—IL-1, IL-6, tumor necrosis factor alpha—TNF-α), chemokine production (monocyte chemoattractant protein 1—MCP-1), adhesion molecule expression (vascular cell adhesion molecule-1—VCAM-1, intercellular adhesion molecular-1—ICAM-1), and monocyte recruitment and differentiation into M1 macrophages [20,21]. These processes also facilitate the migration of T- and B-lymphocytes, dendritic cells (DCs), and vascular smooth muscle cells (VSMCs) into the intima, further amplifying vascular inflammation [1,21]. In this pro-inflammatory environment, endothelial nitric oxide synthase (eNOS) becomes uncoupled, leading to peroxynitrite formation, reduced glutathione scavenging capacity, and decreased nitric oxide (NO) availability, thereby exacerbating endothelial dysfunction [20,21].

OxLDL uptake by macrophages leads to foam cell formation. Foam cells and DCs contribute to the activation of the NOD-like receptor protein 3 (NLRP3) inflammasome, leading to caspase-1 activation and release of IL-1β and IL-18, thereby sustaining a pro-inflammatory milieu [20,21]. VSMCs migrating into the intima not only contribute substantially to foam cell formation (accounting for up to 50% of the total population) but also acquire a pro-atherogenic phenotype by upregulating scavenger receptors, taking up modified lipoproteins, and accumulating cholesteryl esters [20,21,22].

As lesions progress, plaques develop a lipid-rich necrotic core and a fibrous cap composed of VSMCs and extracellular matrix, while VSMC transdifferentiation into osteoblast-like cells contributes to calcification through the deposition of mineralized matrix [20,21]. In advanced stages, the fibrous cap thins due to VSMC apoptosis and metalloproteinase activity and becomes infiltrated by immune cells, increasing rupture risk [23]. Rupture exposes necrotic material, activating platelets and coagulation and potentially causing ischemic cardiomyopathies, MI, or stroke [20,24], although thrombi may remain clinically silent [21]. Lipid-lowering therapy can reduce inflammation and stabilize plaques, preserving blood flow [20].

These interconnected mechanisms collectively define the pathophysiological framework of atherosclerosis and represent key targets for redox and inflammatory modulation, including pathways influenced by Se (see Section 3).

### 2.1. Cellular Senescence

Cellular senescence, originally described more than fifty years ago as a process limiting cell proliferation in vitro, is now widely recognized for its central role in tumor suppression [25]. It is currently defined as a state of irreversible cell cycle arrest in most division-competent cells and has historically been viewed as a protective mechanism that prevents the propagation of damaged DNA, thereby reducing cancer risk [25,26].

Telomere shortening and DNA damage are major drivers of cellular senescence and aging, as progressive telomere attrition—resulting from impaired telomerase activity—eventually triggers DNA damage responses (DDR) when critical length thresholds are reached [27,28,29]. DDR activation induces cell cycle arrest through p21 and p16 signaling pathways, thereby stabilizing the senescent phenotype [26]. DDR activation induces cell cycle arrest through p21 and p16 signaling pathways, thereby stabilizing the senescent phenotype [26].

Prolonged oxidative stress, driven by excessive reactive oxygen species (ROS) production and impaired antioxidant defenses, disrupts redox homeostasis and contributes to cellular damage [26]. This imbalance is exacerbated by reduced activity of nuclear factor erythroid 2–related factor 2 (Nrf2), a key regulator of antioxidant responses, leading to further ROS accumulation and mitochondrial dysfunction [30,31].

Senescent cells also exhibit profound mitochondrial alterations, including impaired respiratory capacity and increased ROS generation, resulting in reduced oxidative phosphorylation, amplifying DNA damage and reinforcing a self-perpetuating cycle of oxidative stress and senescence [32,33,34]. Additionally, metabolic changes such as reduced NAD^+^/NADH ratios activate AMP-activated protein kinase signaling, further stabilizing cell cycle arrest pathways through p53 and p16 regulation [32].

Telomere integrity and mitochondrial biology are reciprocally linked: telomere dysfunction represses peroxisome proliferator-activated receptor gamma coactivator 1α/β, impairing mitochondrial biogenesis and increasing ROS production, which further damages mitochondrial DNA and accelerates telomere shortening, sustaining a self-perpetuating cycle of cellular damage [34].

A hallmark of senescent cells is the senescence-associated secretory phenotype (SASP), characterized by senescent-associated β-galactosidase (SA-β-gal) activity and the coordinated release of pro-inflammatory cytokines (e.g., IL-1β, IL-6, TNF-α, transforming growth factor beta—TGF-β), chemokines, and matrix-remodeling enzymes such as metalloproteinases (MMP-1, MMP-3), which collectively contribute to chronic low-grade inflammation and tissue remodeling [26,35,36,37]. Multiple pathways, including mammalian target of rapamycin (mTOR), NF-ĸB, and p38/mitogen-activated protein kinase (p38/MAPK), sustain the SASP and promote survival and immune resistance of senescent cells [3,37].

Senescent cells exhibit characteristic morphological changes, including enlarged and flattened shapes with increased cytoplasmic vacuolization, and display a marked resistance to apoptosis, contributing to their persistence within tissues [26]. Apoptosis is typically anti-inflammatory and is followed by efferocytosis, in which apoptotic cells are engulfed and cleared by phagocytes, preventing activation of damage-associated molecular patterns and inflammation [38]. Phagocytes also release anti-inflammatory cytokines such as TGF-β and IL-10, contributing to inflammation resolution [38]. In contrast, senescent cells develop a pro-inflammatory and immunogenic phenotype and can persist unless cleared by the immune system or undergoing necrotic death [38].

**Figure 2:**
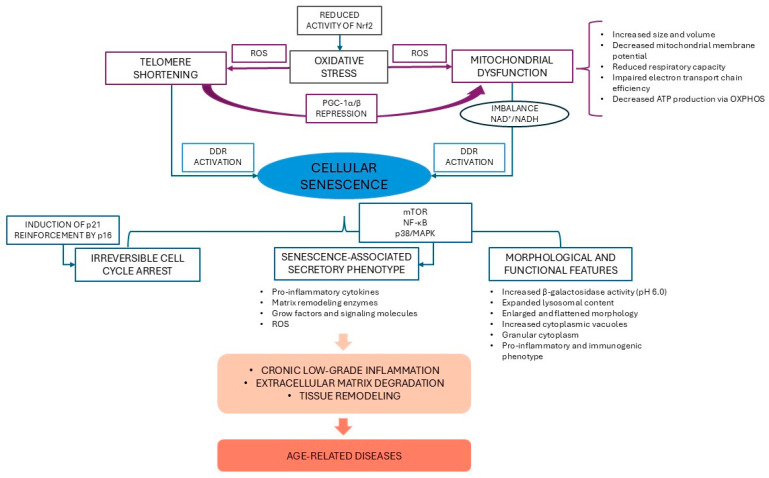
Molecular mechanisms linking telomere dysfunction, oxidative stress, and mitochondrial impairment to cellular senescence and age-related diseases. Abbreviations: DDR: DNA damage response; mTOR: mammalian target of rapamycin; Nrf2: nuclear factor erythroid 2–related factor; NF-ĸB: nuclear factor kappa-light-chain-enhancer of activated B cells; OXPHOS: oxidative phosphorylation; p38/MAPK: p38/mitogen-activated protein kinase; PGC-1α/β: proliferator-activated receptor gamma coactivator 1α/β; ROS: reactive oxygen species.

Although initially attributed to telomere-dependent replicative senescence, multiple additional forms have been identified, including oncogene-induced senescence [38,39]. Oncogene-induced senescence is a complex program that activates tumor suppressive responses, but its SASP may also promote tumor progression through secretion of pro-inflammatory cytokines, C-X-C motif chemokine ligand 1 and vascular endothelial growth factor (VEGF), enhancing angiogenesis and supporting neighboring cell growth [26,40]. Overall, while senescence provides a short-term barrier against malignant transformation, its long-term persistence, driven by chronic inflammation, contributes to tumor progression and multiple age-related diseases, including cardiovascular and cerebrovascular diseases, neurodegenerative disorders, fibrotic pulmonary diseases, hepatic steatosis, and metabolic dysfunction [4,41,42] (Figure 2).

### 2.2. The Role of Cellular Senescence in Atherosclerosis

**Figure 3:**
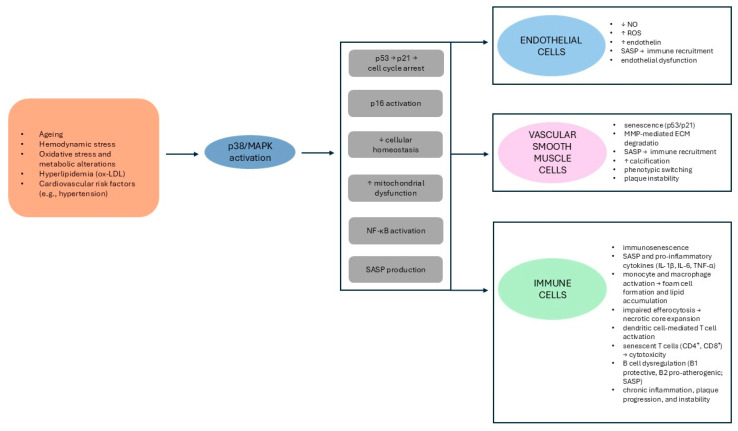
Integrated role of cellular senescence in vascular and immune dysfunction during atherosclerosis (see text for details). Arrows indicate increase (↑) and decrease (↓). Abbreviations: ECM: extracellular matrix; LDL: low-density lipoprotein; MMP: metalloproteinase; NF-ĸB: nuclear factor kappa-light-chain-enhancer of activated B cells; NO: nitric oxide; oxLDL: oxidized low-density lipoprotein; p38/MAPK: p38/mitogen-activated protein kinase; ROS: reactive oxygen species; SASP: senescence-associated secretory phenotype.

Accumulating evidence indicates that cellular senescence is a major driver of atherosclerosis, linking aging-related processes with vascular dysfunction and chronic inflammation [3,4]. Multiple stressors—including mechanical forces, oxidative imbalance, and metabolic alterations—as well as cardiovascular risk factors such as hypertension, hyperlipidemia, obesity, diabetes, and smoking, contribute to its induction in vascular and immune cells [3,43]. Many of these conditions are age-associated, highlighting the close interplay between aging, senescence, and atherosclerosis [3]. Below, we summarize the contribution of senescent vascular and immune cells to atherosclerosis (Figure 3).

#### 2.2.1. Vascular Endothelial Cells

Vascular ECs are among the earliest cells to exhibit aging due to continuous exposure to circulating stressors and disturbed flow at arterial branches, where low and oscillatory shear stress increases mechanical burden and endothelial turnover [44]. These conditions promote telomere attrition, DDR activation, p53–p21 signaling, and replicative senescence [3]. Pro-inflammatory and pro-atherogenic stimuli (e.g., TNF-α, hydrogen peroxide, oxLDL) further impair protein kinase B signaling, reducing telomerase activity and accelerating EC aging [45,46].

Metabolic disorders such as obesity, hyperlipidemia, and diabetes exacerbate EC senescence [3]. In particular, hyperglycemia-induced downregulation of sirtuin-1 (SIRT1) accelerates endothelial aging [47]. SIRT1 regulates DNA repair and oxidative stress responses via Nrf2 and Forkhead box protein O3 and modulates antioxidant enzymes through epigenetic mechanisms [48,49]. Reduced SIRT activity increases mitochondrial dysfunction and oxidative stress [50].

Senescent ECs exhibit elevated ROS, reduced NO bioavailability, and increased peroxynitrite formation, impairing shear stress-mediated vasodilation [51]. Combined with enhanced p53 activity and endothelin production, this suppresses angiogenic signaling (VEGF, fibroblast growth factor, and hypoxia-inducible factor) and promotes endothelial dysfunction [44,52,53]. These cells also acquire a prothrombotic phenotype via upregulation of plasminogen activator inhibitor-1 and thromboxane A2 [44,51].

Finally, senescent ECs develop a SASP enriched in cytokines (IL-1, IL-6, IL-8, TNF-α), chemokines (MCP-1, CCL11), and adhesion molecules (ICAM-1), facilitating immune cell recruitment and vascular inflammation [3,44] (Figure 3).

#### 2.2.2. Vascular Smooth Muscle Cells

VSMCs are key contributors to all stages of atherosclerosis [54,55]. Senescent VSMCs, found in aged vessels and advanced plaques, arise from telomere-dependent replicative senescence and stress-induced premature senescence triggered by Angiotensin II (Ang II), inflammation, and oxidative stress [44,56,57].

Ang II, the principal effector molecule of the renin–angiotensin–aldosterone system and a key mediator in the pathophysiology of CVD [58,59], promotes endothelial dysfunction, VSMC proliferation, inflammation, and pathological remodeling via p53/p21 activation; p21 inhibition attenuates these effects [59,60]. It also stimulates TGF-β signaling, NF-κB activation, ROS production, and MMP activity while reducing NO bioavailability [61]. MMP-2 and MMP-9 contribute to extracellular matrix degradation, calcification, and plaque instability [44,57], whereas TGF-β drives collagen I/III deposition, altering fibrous cap stability [62,63]. Imbalance between collagen synthesis and degradation leads to cap thinning and increased rupture risk [63].

Senescent VSMCs exhibit SASP-associated cytokines (IL-1, IL-6, IL-8, TNF-α) and increase ROS production due to reduced antioxidant defenses [55,57]. They also shift toward an osteogenic phenotype, expressing osteopontin, osteoprotegerin, runt-related transcription factor 2, bone morphogenetic protein 2, and alkaline phosphatase, promoting vascular calcification and stiffness [44,55]. VSMCs may promote survival through autophagy, a lysosome-regulated process that degrades and recycles unnecessary or dysfunctional components to maintain intracellular homeostasis and energy balance [54,55,57,64]. Autophagy modulates VSMC survival, with mTOR activation promoting senescence and autophagy inhibition [65]. Moderate levels of oxLDL induce autophagy, whereas higher concentrations impair flux and accelerate premature senescence, underscoring the inverse relationship between autophagy and senescence in atherosclerosis [54,55].

#### 2.2.3. Immune Cells

Immunosenescence contributes to atherosclerosis through coordinated alterations across multiple immune cell populations, including monocytes, macrophages, DCs, and lymphocytes, which collectively sustain chronic inflammation and plaque progression [66].

Monocytes, particularly CD14/CD16 subsets, are central to lesion formation and exhibit hallmarks of cellular senescence [67,68,69], that enhance their pro-inflammatory activity, including increased cytokine production (e.g., TNF-α, IL-6, IL-8) and upregulation of adhesion molecules and chemokine receptors that promote endothelial adhesion and vascular recruitment [70,71].

Senescent macrophages contribute to atherosclerosis progression by promoting lipid accumulation, foam cell formation, and necrotic core expansion, partly due to impaired efferocytosis [3,37]. In parallel, they acquire a pro-inflammatory phenotype characterized by increased expression of cytokines and matrix-degrading enzymes (e.g., MMPs), which contribute to extracellular matrix degradation and plaque instability [69,72,73,74].

DCs accumulate within the arterial intima during aging and atherosclerosis and display altered immune function, including reduced T-cell interaction capacity and increased production of pro-inflammatory mediators [75,76,77]. They can also contribute to foam cell formation and promote T-cell activation, sustaining local immune responses through cytokine production and metabolic reprogramming [78,79,80].

T cells are key drivers of atherosclerosis, with different subsets contributing to disease progression [3,81]. Senescent CD4+ and CD8+ T cells exhibit enhanced pro-inflammatory and cytotoxic activity, partly driven by p38/MAPK signaling and characterized by increased production of interferon-γ and other effector molecules [81,82,83]. T cells also display metabolic and functional alterations, including increased glycolytic activity and cytotoxic mediator release (e.g., granzyme B and perforin), which further amplify vascular damage [84,85].

B cells exert both protective and pro-atherogenic effects, depending on the subset involved [86,87]. While B1 cells produce atheroprotective IgM antibodies, B2 cells contribute to disease progression through IgG production and pro-inflammatory signaling [87,88,89]. Senescent B-cell subsets further display SASP features, including increased cytokine release and enhanced recruitment to inflamed vascular sites, mediated by NF-κB and p38/MAPK signaling pathways [89,90].

Collectively, these alterations highlight the central role of immunosenescence in driving vascular inflammation and plaque progression, representing key targets for redox modulation, including pathways influenced by selenium.

## 3. Selenium, Cellular Senescence and Atherosclerosis

### 3.1. Selenium: An Overview

Se, a metalloid belonging to the same group as oxygen and sulfur, is a well-established essential trace element in humans, where it serves as a key structural component of enzymes with antioxidant, anti-inflammatory and immunomodulatory properties [9,91]. The total amount of Se in humans ranges between 3–20 mg, with intake primarily derived from food sources and, to a much lesser extent, from water and atmospheric inputs [9,92]. Although cereals represent the main dietary source of Se in terms of bioavailability, plant-based foods generally contain lower Se levels (0.01–0.55 μg/g) compared with animal-based foods (0.08–0.7 μg/g) [92]. This difference largely reflects the variability of Se concentrations in soils, which range from 0.005 to 3.5 mg/kg depending on the country and region, even within the same country, with a global average soil Se concentration of approximately 0.32 mg/kg [93,94]. Soil Se bioavailability is influenced not only by physicochemical properties (e.g., pH, redox status, soil moisture, organic matter content, microbial activity) but also by its chemical speciation [95]. In soil solution, the oxyanions (Se(VI)), predominant in well-aerated alkaline soils, and selenite (Se(IV)), more abundant in neutral to acidic soils, represent the most mobile and bioavailable forms for plant uptake [93,95]. Their interconversion largely depends on soil pH and redox potential, with selenite frequently bound to iron hydroxides in acidic soils [91,93,95]. Organic Se species—including Se-containing proteins, metabolites, and Se covalently bound to soil organic matter—constitute an additional plant-available pool through direct uptake (e.g., SeCys, selenomethionine—SeMet, and methylselenocysteine), oxidation to inorganic oxyanions, or mobilization during organic-matter turnover [93]. In contrast, insoluble elemental Se (Se^0^) and selenide (Se^2−^), which predominate under reducing conditions, are poorly available to plants [95]. In foods, selenoamino acids exhibit higher bioavailability than inorganic Se species [92]. SeMet, synthesized exclusively by plants and fungi, reaches 95–98% bioavailability in plant-derived matrices, whereas SeCys is the predominant chemical form in animal-based foods, reflecting its direct incorporation into selenoproteins [9,92].

Following absorption in the duodenum, Se is distributed to tissues with different affinities, with the liver and skeletal muscle each accounting for approximately 30% of total body stores [9,96]. In enterocytes, dietary Se is incorporated into selenoproteins in the form of SeCys, which is essential for their biological activity [9,92]. SeCys, the 21st naturally occurring amino acid, lacks a cognate aminoacyl-tRNA synthetase in humans and therefore requires a specialized biosynthetic pathway for its incorporation into selenoproteins [92]. SeCys is inserted into SELENOP, a glycoprotein containing 10 SeCys residues and accounting for up to 40% of total circulating Se, thus serving as the main Se transport protein to deliver Se to other cells [92]. To date, 25 selenoproteins have been identified in humans [92]. Distributed across different organs, most selenoproteins, including GPx and TrxR, play essential roles in antioxidant defense [92]. Their antioxidant activity has also been related to chemopreventive mechanisms and a reduced cancer risk [97]. Se supplementation enhances both innate and adaptive immune responses, with immunostimulatory effects detectable also in individuals with adequate Se status [10,92]. SELENOP, SELENOW, and GPx4 are among the most abundantly expressed selenoproteins in the brain, underscoring their importance in preserving neural function; notably, the brain contains one of the largest Se reservoirs in the body—second only to the liver—and this concentration is maintained under conditions of dietary Se depletion [92,97]. Beyond its transport function, SELENOP exhibits redox properties that protect endothelial cells from oxidative injury, while other selenoproteins—such as GPx3, SELENOS, and SeAlb—have been associated with cardiovascular health through their roles in regulating oxidative stress and inflammation [97].

The World Health Organization and the Food and Agriculture Organization recommend a daily Se intake of 55–60 μg for adults; however, global dietary guidelines are not standardized, and recommendations vary across countries and organizations [7,9,98]. The European Food Safety Authority has set an adequate intake of 70 μg/day and a tolerable upper intake level of 255 μg/day for adults [99,100].

Se exerts both beneficial and adverse effects depending on its concentration [101]. Chronic high-level Se exposure—resulting from over supplementation, frequent consumption of Se-rich foods, or occupational exposure (e.g., Se processing plants)—can lead to selenosis, a condition characterized by nail brittleness, hair loss, fatigue, and skin lesions [101,102]. As the condition progresses, neurological impairment may develop, including cognitive decline and paralysis, and in severe cases, it can be fatal [102]. The toxic effects of excessive Se exposure are thought to arise from disruption of cellular redox balance and the paradoxical induction of oxidative stress [103]. Although Se is an essential component of antioxidant selenoproteins, supraphysiological levels may promote the generation of ROS [104], impair mitochondrial function [105], and alter the activity of redox-sensitive signaling pathways involved in inflammation [106] and apoptosis [103]. In addition, excess Se may interfere with sulfur-containing amino acid metabolism [107] and contribute to metabolic dysregulation, including alterations in lipid and glucose homeostasis [108,109]. Moreover, high Se intake has been associated with renal dysfunction, increased cardiovascular risk, MI, and heart failure, highlighting that the beneficial effects of Se occur within a narrow range of plasma concentrations [9,110,111,112]. These observations support the existence of a U-shaped relationship between Se status and health outcomes, in which both Se deficiency and Se excess may exert detrimental biological and clinical effects.

### 3.2. Selenium and Cardiovascular Disease in Human Studies: Focus on Atherosclerotic Cardiovascular Diseases

Despite inconsistent findings from early observational studies and Se supplementation trials, an increasing body of evidence supports a role for Se in the optimal functioning of the cardiovascular system [113]. In this context, a recent meta-analysis of 13 observational studies and randomized controlled trials (RCTs) reported a 15% reduction in CVD incidence per 10 µg/L increase in blood Se concentration, together with a statistically significant nonlinear dose–response relationship between blood Se levels and CVD mortality [114]. The lowest risk was observed at Se concentrations between 30 and 35 µg/L, whereas CVD risk increased at concentrations above 300 µg/L, indicating that Se may exert protective effects only within a physiologically optimal range for both CVD incidence and mortality [114].

In one of the earliest comprehensive assessments, Flores-Mateo and colleagues investigated the association between Se and CHD by conducting a meta-analysis of observational studies and RCTs [115]. In the analysis of observational studies (14 prospective cohort and 11 case–control studies), the pooled relative risk (RR) comparing the highest with the lowest category of blood Se concentration was 0.85 (95% Confidence Interval—CI: 0.74–0.99) in cohort studies and 0.43 (95% CI: 0.29–0.66) in case–control studies. Moreover, dose–response analyses indicated a 24% reduction in CHD risk associated with a 50% increase in Se concentration [115]. By contrast, the meta-analysis of RCTs (six trials: two using Se alone and four using Se in combination with vitamins or minerals; Se doses of 75, 100, or 200 µg/day) showed a lower, but not statistically significant, risk of CHD with Se supplementation compared with placebo (pooled RR = 0.89; 95%CI: 0.68–1.17) [115]. It should be noted, however, that the inverse associations observed in observational studies require confirmation after adequate adjustment for potential confounders, including other dietary antioxidants (such as vitamin E, folate, and β-carotene), overall dietary patterns, and Se intake from foods or supplements, as well as residual confounding related to socioeconomic status, educational level, and other cardiovascular risk factors [115]. By contrast, the limited evidence from randomized trials may, at least in part, be explained by the relatively small number of participants and by substantial heterogeneity across studies, related to differences in baseline Se status, treatment duration, and intervention regimens, including combined supplementation strategies [115]. Zhang et al. confirmed these findings when the outcome was expanded to include total CVD [116]. Specifically, the meta-analysis of 16 prospective studies (*N* = 35,607 participants and 4421 incident CVD cases) showed a significant inverse association with CVD risk within a relatively narrow range of blood Se concentrations (55–145 µg/L), which became null at concentrations exceeding 145 µg/L [116]. Conversely, the meta-analysis of 16 RCTs (37,572 participants) performed by the same authors found no significant effect of oral Se supplementation (median dose: 100 µg/day) on CVD risk over follow-up periods ranging from 6 to 114 months [116]. Indeed, although beneficial effects of Se may be more likely in deficient populations, adequate or high Se status may be associated with neutral or even adverse effects [116]. Moreover, the authors could not exclude potential changes in compliance among trial participants or heterogeneity in Se concentration responses following supplementation [116,117].

In a subsequent meta-analysis including 16 RCTs and a total of 43,998 participants, Ju et al. observed a non-significant trend toward reduced CHD mortality among individuals receiving Se supplementation compared with controls (Odds Ratio—OR = 0.88; 95%CI: 0.76–1.02; *p* = 0.087) [118]. Consistently, Se supplementation was not significantly associated with total cholesterol, LDL or high-density lipoprotein cholesterol levels [118]. By contrast, the intervention was associated with significantly lower serum levels of C-reactive protein, a major inflammatory marker and a well-established risk factor for CHD that is directly correlated with both coronary and peripheral atherosclerosis [119,120], as well as with increased GPx activity [118]. Accordingly, Se supplementation appears to attenuate inflammation and oxidative stress, despite the absence of significant effects on lipid profiles or CHD mortality [118]. The lack of association with CHD mortality, in particular, may reflect the chronic and progressive nature of the disease, characterized by a high burden of complications and fatal events; thus, short-term Se supplementation may be insufficient to reduce mortality, while still being effective in improving inflammatory and pro-oxidant status [118]. Additionally, given that in two trials participants receiving placebo exhibited higher mortality than those receiving combined Se and coenzyme Q10 supplementation, the combination of Se with other supplements may have a greater impact on reducing CHD mortality than Se alone [118]. This notion is further supported by a meta-analysis of 43 RCTs by Jenkins et al., which showed that Se supplementation was associated with reduced cardiovascular and all-cause mortality only when combined with antioxidant mixtures (e.g., vitamin A, vitamin C, vitamin E, β-carotene, zinc, copper) (RR = 0.77, 95%CI: 0.62–0.97; RR = 0.90, 95%CI: 0.82–0.98) [121]. On the other hand, neither Se alone nor antioxidants alone had significant effects, suggesting that Se-related effects are finely balanced and may depend on synergistic antioxidant interactions [121]. A recent systematic review and meta-analysis of 884 RCTs evaluating the effects of 27 different micronutrients (median duration intervention: 3 years) on CVD outcomes in a total of 883,627 participants found a moderate beneficial effect of Se on dyslipidemia [122]. However, no significant associations were observed with the incidence of CHD (RR = 0.89, 95%CI: 0.64–1.23), MI (RR = 0.87, 95%CI: 0.59–1.30), or CVD mortality (RR = 0.97, 95%CI: 0.83–1.14) [122]. Importantly, several intervention trials had very short durations (<1 month), and the effects of antioxidant mixtures were not evaluated, raising the possibility that the overall cardioprotective effects of Se supplementation may have been underestimated [122].

A meta-analysis of 12 observational studies (10 cohort and 2 case–control studies), including a total of 25,667 individuals, confirmed that low Se levels were associated with an increased risk of both all-cause and CVD mortality [123]. When comparing the lowest with the highest category of circulating Se levels, the pooled RRs were 1.36 (95%CI: 1.18–1.58) for all-cause mortality and 1.35 (95%CI: 1.13–1.62) for CVD mortality [123]. In contrast, the association with CHD mortality, assessed in four studies, was not statistically significant (pooled RR = 1.43, 95%CI: 0.93–2.19) [123]. This lack of significance may be partly explained by the reliance on a single baseline measurement of Se, which could have led to exposure misclassification and dilution of risk estimates across Se level categories [123]. On the other hand, because high-normal Se concentrations (>150 µg/L) may be associated with an increased risk of mortality [124], the inclusion of the highest Se category as the reference group may have led to an underestimation of the association between low Se levels and CVD mortality [123]. Consistently, the protective effects of low Se status on CVD and all-cause mortality were more evident in Europe and Asia, regions characterized by populations with low Se intake (<50 µg/day) or low Se status (serum concentrations <100 µg/L) [114,123]. More recently, Yang et al. examined differences in Se levels between patients with CHD or MI and healthy controls within a meta-analysis including 38 studies and 25 cohort populations [125]. Both MI and CHD patients exhibited significantly lower blood Se levels (Standard Mean Difference—SMD = −3.64, 95%CI: −4.43, −2.85; SMD = −0.47, 95%CI: −0.67, −0.28) compared to healthy controls, supporting the hypothesis that low Se status may be associated with an increased risk of atherosclerotic cardiovascular disease (ASCVD) [125]. Circulating Se levels also demonstrated a favorable diagnostic performance for MI, with relatively high sensitivity (77.27%) and specificity (72.73%) [125]. Based on data from the U.S. National Health and Nutrition Examination Survey (NHANES) 2011–2016, a cross-sectional study involving 5101 participants reported a strong L-shaped association—rather than a U-shaped relationship as previously described by Kuria et al. [114]—between blood Se concentrations and the prevalence of CHD [126]. In addition, participants in the second, third, and fourth quartiles of blood Se concentration exhibited significantly lower odds of CVD morbidity compared with those in the lowest quartile, consistent with a dose–response pattern (OR = 0.71, 95%CI: 0.53–0.96, *p* = 0.024; OR = 0.73, 95%CI: 0.55–0.99, *p* = 0.041; and OR = 0.74, 95%CI: 0.55–0.98, *p* = 0.038, respectively), indicating that even the highest Se quartile was associated with a protective effect [126]. However, these findings should be interpreted with caution, as the cross-sectional study design does not allow causal inferences regarding the relationship between Se status and CVD. Cui et al. were the first to assess the relationship between Se status, Se-related biomarkers, and CVD mortality in a meta-analysis of population-based studies (*N* = 9; 41,548 participants) [127]. In pooled analyses, each standard deviation increase in Se/SELENOP concentration was associated with an 11% reduction in CVD mortality across nine studies including 41,548 participants (RR = 0.89, 95%CI: 0.84–0.94) [127]. This finding contrasts with the results reported by Kuria et al. [114], who observed no significant association between a 10 μg/L increase in blood Se concentration and CVD mortality (RR = 0.93, 95%CI: 0.83–1.05); however, their meta-analysis was not restricted to population-based studies [127].

Of interest, Liang et al. recently explored the relationship between dietary Se intake and the risk of CVD in U.S. adults using data of 39,372 participants from the NHANES 2003–2018 [17]. The overall prevalence of CVD was 8.57% and progressively decreased across increasing tertiles of dietary Se intake (11.10% in the lowest tertile vs. 6.75% in the highest tertile) [17]. Furthermore, after adjustment for potential confounders, a significant inverse association between dietary Se intake and CVD risk was observed when comparing the highest tertile with the reference category (OR = 0.73, 95%CI: 0.63–0.86, *p* < 0.0001) [17]. Similarly, higher dietary Se intake was associated with a significantly reduced risk of CHD (OR = 0.78, 95%CI: 0.64–0.95, *p* = 0.01; third vs. first tertile) and ASCVD (OR = 0.85, 95%CI: 0.74–0.98, *p* = 0.02; second vs. first tertile) [17]. Notably, the relationship between dietary Se intake and both CVD and ASCVD followed a significant non-linear pattern (*p* = 0.002), with risk decreasing beyond an inflection point at 135.28 µg/day and subsequently increasing at higher intakes, suggesting a potential increased risk of adverse outcomes at excessive Se intakes [17]. Subgroup analyses further showed that the inverse association between higher dietary Se intake and ASCVD risk was particularly evident among females, individuals younger than 60 years, and those with obesity or hypertension [17]. Moreover, hypertension status significantly modified the association between dietary Se intake and ASCVD risk (*p* for interaction = 0.034) [17]. Despite these relevant findings, it is important to note that the cross-sectional study design cannot capture potential variations in dietary patterns over time, and such changes may influence the observed relationship between Se intake and CVD [17]. In addition, reliance on self-reported CVD outcomes may have introduced recall bias [17] (Table 1).

Taken together, human studies provide divergent evidence depending on study design. While multiple observational studies’ meta-analyses consistently suggest an inverse or non-linear association between Se status and ASCVD risk, RCTs largely fail to demonstrate significant benefits on reduced CHD/CVD events or mortality with Se supplementation alone. These discrepancies highlight the importance of baseline Se status, the identification of an optimal dose—possibly in combination with other beneficial antioxidants—and adequate study duration when designing and implementing RCTs. Furthermore, the chemical form of Se supplementation may influence circulating Se concentrations, with SeMet being among the most effective organic selenocompounds for improving Se status; this aspect may contribute to an underestimation of the beneficial effects of Se supplementation in some trials.

**Figure 4:**
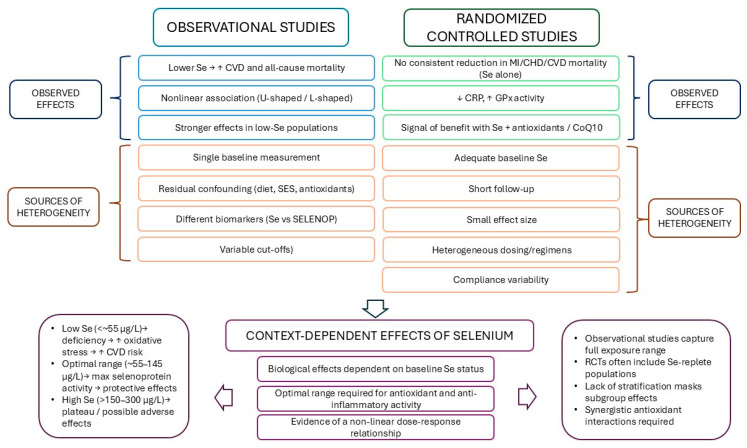
Conceptual framework summarizing evidence from observational and randomized studies on selenium and cardiovascular risk. Arrows indicate increase (↑) and decrease (↓). Abbreviations: CHD: coronary heart disease; CoQ10: coenzyme Q10; CRP: C-reactive protein; CVD: cardiovascular disease; GPx: glutathione peroxidase; MI: myocardial infarction; RCT: randomized controlled trial; Se: selenium; SES: socioeconomic status; SELENOP: selenoprotein P.

On the other hand, observational studies are inherently subject to potential bias. In particular, reliance on a single baseline Se measurement may lead to exposure misclassification, while the observational nature of these studies cannot fully rule out residual confounding (diet quality, antioxidant intake, socioeconomic status). Causality remains uncertain, as observational associations may be influenced by residual and reverse causation. In addition, substantial heterogeneity in the cut-off values used to define Se status across studies limits the ability to identify precise optimal circulating Se levels in the clinical setting. In addition, as summarized in Figure 4, the relationship between Se status and cardiovascular outcomes appears to be nonlinear and strongly dependent on baseline Se levels, which may account for the heterogeneity between observational and interventional studies. Future trials should be stratified by baseline Se status, focusing on deficient or low-status individuals, to clarify whether supplementation benefits are restricted to specific subgroups. There is a need for long-term RCTs with adequate duration and statistical power, given the chronic and progressive nature of atherosclerosis and CHD.

### 3.3. Cellular and Molecular Mechanisms of Selenium in Preventing Atherosclerosis

**Figure 5:**
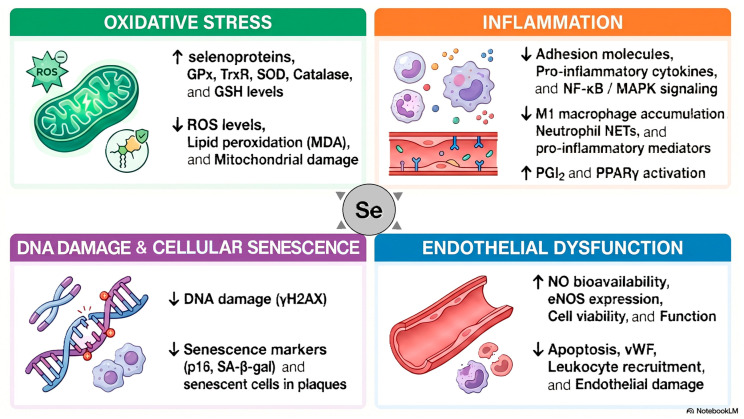
Selenium mechanisms in contrasting key processes of atherosclerotic disease. Arrows indicate changes in the parameter: increase (↑) and decrease (↓). Image partially generated with Notebook LM. Abbreviations: eNOS: endothelial nitric oxide synthase; GPx: glutathione peroxidase; GSH: glutathione; MAPK: mitogen-activated protein kinase; MDA: malondialdehyde; NETs: neutrophil extracellular traps; NF-κB: nuclear factor kappa-light-chain-enhancer of activated B cells; NO: nitric oxide; PGI2: prostaglandin I2; PPARγ: peroxisome proliferator-activated receptor-gamma; ROS: reactive oxygen species; SA-β-gal: senescence-associated beta-galactosidase; Se: selenium; SOD: superoxide dismutase; TrxR: thioredoxin reductase; vWF: von Willebrand factor.

As previously mentioned, when present in appropriate concentrations, Se exerts its beneficial effects, playing a key role in protecting against atherosclerosis. Se contribution is summarized in Figure 5 and was proved by several in vitro and in vivo studies, hereby described, that demonstrated how this trace element can influence multiple and interconnected pathways of atherosclerotic cascade, including maintenance of redox balance, control of inflammation, and prevention of DNA damage, cellular senescence and endothelial dysfunction.

#### 3.3.1. Selenium in Contrasting Oxidative Stress

The status of oxidative stress, characterized by the imbalance between oxidant levels (in particular ROS) and ineffective antioxidant defense, is strongly linked to atherosclerosis. This association is due to the enhanced production of these reactive species, caused by all established cardiovascular risk factors, including hypercholesterolemia, hypertension, diabetes mellitus, and smoking. As described in Section 2, vascular regions presenting this condition of oxidative stress provoke a disturbance in blood flow, thus becoming preferential sites for atherosclerotic plaque formation [128].

In this scenario, every contribution to antioxidant defenses can exert an essential role. Se directly participates in the suppression of oxidative stress, by associating with selenoproteins and scavenging damaging oxidant species, as well as bolstering endogenous antioxidant defense systems [129]. Indeed, several studies demonstrated that Se not only takes part in constituting selenoproteins but also increases levels and activity of multiple antioxidant mediators. In 1998, Rosenblat & Aviram provided evidence that Se increases GSH content and GPx activity by 2-fold in murine macrophage-like cells, resulting in a 30% reduction in macrophage-mediated LDL oxidation, a key early event in atherogenesis [130]. Further in vitro studies employing the same model demonstrate that Se-containing organic compounds—such as diphenyl diselenide and disubstituted diaryl diselenides—attenuate ROS generation, foam cell formation, NF-κB activation, and mitochondrial dysfunction through GPx- and TrxR-mimetic activities [131,132]. In rat VSMCs, pre-treatment with sodium selenite prevents the oxidative stress induced by oxysterol, preserving TrxR and GPx expression levels, sustaining the activity of this latter and superoxide dismutase (SOD) and maintaining the total antioxidant capacity, with the inhibition of ROS generation [133]. In human umbilical vein endothelial cells, HUVECs, Se pre-treatment, in the form of sodium selenite, confers protection against chemically induced oxidative damage, by stimulating TrxR expression and increasing the activity of this enzyme, as well as that of two other selenoproteins, GPx1 and GPx4, by 3–4 fold [134]. More recently, Se was incorporated into an integrated cascade nanozyme (MSe_1_), with SOD- and GPx-like activities, resulting in decreased ROS levels in HUVECs and RAW264.7 macrophages [135].

The first in vivo evidence of the antioxidant properties of Se was observed within 90 s in rabbits, in a synergic combination with vitamin E [136,137], and in hamsters in association with glutathione [138]. More recent studies have better characterized the actions of this element. Nanoscale Se showed protective effects against mitochondrial oxidative damage and apoptosis in hyperhomocysteinemia conditions, by hampering downregulation of GPx1 and GPx4 in rats’ vascular ECs [139]. Similarly, the human umbilical vein cell line, EA.hy926, when pre-treated with Se nanoparticles (SeNPs), is less susceptible to hydrogen peroxide, as evidenced by its lower levels of malondialdehyde (MDA), a key product of lipid peroxidation and marker of oxidative stress, and increased activity of GPx and SOD. Increased activity of these enzymes, along with catalase, following SeNP treatment, was confirmed in vivo in apolipoprotein E-deficient (ApoE^−/−^) mice fed a high-fat diet. In this model, SeNPs also upregulate the expression of six additional selenoproteins, TrxR1, TrxR2, SELENOP, SELENOR, SELENOS, and 15-kDa selenoprotein, resulting in reduced serum and hepatic MDA levels [140]. Analogous results were reported by Xiao et al. using sodium selenite and chitosan-stabilized SeNPs [141]. Another study conducted in spontaneously hypertensive rat models demonstrated that Se supplementation improvess the overall redox status of the aortic wall by enhancing GPx1 activity and reducing lipid peroxidation, eNOS expression, and markers of advanced glycation end products [142]. Furthermore, a recent study showed that a Se-containing confined cascade nanozyme system exerts potent antioxidant effects in both endothelial cell models and ApoE^−/−^ mice. This effect is mediated by enhanced ROS scavenging through catalytic cascade reactions, leading to reduced intracellular ROS levels, decreased lipid peroxidation, and attenuation of oxidative stress–related vascular damage, thereby supporting the role of Se in maintaining redox homeostasis [143].

#### 3.3.2. Selenium in Regulating Inflammatory Mediators

Atherosclerosis is characterized by a persistent inflammation of the vascular wall, where endothelial activation, leukocyte recruitment, and cytokine production drive lesion development and progression [144].

Remarkably, Se was reported to influence several cellular and molecular mediators of this cascade, thus directly and indirectly mitigating activation and amplification of inflammation. In HUVECs, in inflammatory conditions, Se contrasts the expression of three important adhesion molecules, E-selectin, VCAM-1, and ICAM-1 [145]. In murine RAW 264.7 macrophages, Se promotes the production of 15d-PGJ_2_, a prostaglandin that suppresses pro-inflammatory mediators (IL-6, TNF-α, and NO) and activates peroxisome proliferator-activated receptor gamma, a key regulator of lipid homeostasis that attenuates inflammation and limits plaque development [146]. Additional evidence supporting the role of Se in controlling atherosclerosis-related inflammation is provided by the study of Cao et al., conducted in bovine mammary endothelial cells cultured under Se-deficient conditions. In the absence of Se, arachidonic acid metabolism shifts toward a pro-inflammatory and pro-thrombotic profile, characterized by reduced production of vasodilatory and anti-aggregatory prostacyclin (PGI_2_) and other protective prostaglandins (PGF_2_α, PGE_2_), along with a significant increase in pro-aggregatory thromboxane A_2_ and the pro-inflammatory lipid hydroperoxide 15-HPETE [147]. Moreover, Se can indirectly limit vascular inflammation by upregulating GPx1/GPx4. Indeed, experimental downregulation of these enzymes in ECs leads not only to ROS accumulation but also to increased expression of adhesion molecules (ICAM-1, VCAM-1) and enhanced leukocyte recruitment via activation of the NF-κB and MAPK pathways. In contrast, their upregulation or pharmacological mimicking attenuates these response [18].

The anti-inflammatory properties of Se are further supported by some in vivo studies. In 1992, Meydani et al. were among the first to demonstrate, in an animal model (F344 rats), that Se deficiency reduces aortic PGI_2_ synthesis and shifts the thromboxane B_2_/PGI_2_ ratio toward a pro-thrombotic profile, thereby directly linking dietary Se to vascular prostacyclin production relevant to atherogenesis [148]. Additionally, in male Sprague–Dawley rats, serum Se levels were found to be inversely correlated with cholesterol levels, and Se supplementation not only reduced ROS levels but also downregulated CD36 expression, a scavenger receptor involved in oxLDL uptake, foam cell formation, and vascular inflammation [149]. Consistently, in ApoE^−/−^ mice fed a high-fat diet, SeMet reduces atherosclerotic plaque development by modulating inflammatory cell behavior, as indicated by decreased M1 macrophage accumulation within lesions and reduced neutrophil extracellular trap formation; these effects were also confirmed ex vivo in human neutrophils [150]. In the same mouse model, Xiao et al. demonstrated that both SeNPs and selenite prevent vascular inflammation, as evidenced by reduced levels of pro-inflammatory cytokines (TNF-α, IL-1β, IL-6), decreased NF-κB signaling in the vessel wall, and reduced macrophage infiltration and VSMC migration [148].

Emerging human evidence supports the anti-inflammatory power of Se; in fact, a recent epidemiological study reported that Se concentration is inversely associated with pro-inflammatory markers, such as TNF-α, IL-6, and MCP-1 [151].

#### 3.3.3. Selenium in Preventing DNA Damage and Cellular Senescence

DNA damage accumulating in vascular and resident cells is recognized as a key contributor to the initiation and progression of atherosclerosis, triggering an overwhelmed cellular response that promotes senescence, apoptosis, and plaque instability [152].

Adequate Se status is reported to counteract several hallmarks of aging that are also implicated in atherogenesis, including oxidative DNA damage, telomere attrition, and cellular senescence [129]. Based on these properties, Se—particularly when incorporated into nanozymes—is being investigated as a potential preventive strategy for atherosclerotic disease. Remarkably, Liu et al. developed a MSe_1_ with SOD- and GPx-like activities that exerts antisenescence effects in HUVECs, reducing the levels of γH2AX, a marker of DNA damage, as well as the senescence marker p16, and SA-β-gal activity. Consistently, reduced signals of these markers were also observed in plaque areas of ApoE^−/−^ mice treated with MSe_1_ [135]. Similar findings were reported by Huang et al., who demonstrated that a Se-doped copper formate (Cuf-Se) nanozyme, endowed with SOD- and GPx-like activities, not only scavenges ROS and inhibits foam cell formation but also reduces cellular senescence in HUVECs, as evidenced by decreased γH2AX levels. These effects were confirmed in vivo in ApoE^−/−^ mice, where treatment with Cuf-Se significantly reduced the number of senescent cells in aortic root sections [153].

#### 3.3.4. Selenium in Attenuating Endothelial Dysfunction

Endothelial dysfunction, a hallmark of atherosclerosis, is characterized by the loss of normal endothelial functions, including impaired vasodilation, increased oxidative stress, and a pro-inflammatory and pro-thrombotic state. This altered phenotype creates a permissive environment for lipid accumulation and vascular inflammation, thereby promoting macrophage recruitment and plaque development [154].

An increasing body of evidence indicates that Se can attenuate endothelial dysfunction through multiple mechanisms. In HUVECs exposed to homocysteine as a model of vascular injury, Se was shown not only to reduce apoptosis but also to improve cell viability, preserve NO bioavailability, and mitigate oxidative stress via activation of the Akt pathway, collectively contributing to the restoration of endothelial function. These protective effects were further confirmed in male Sprague–Dawley rats, which exhibited increased plasma NO levels and reduced levels of von Willebrand factor, a marker of endothelial damage, following selenium supplementation [155]. Consistently, nanoscale Se protects both ECs and hyperhomocysteinemic rats from vascular damage [147].

Huang et al. further demonstrated the protective role of Se in Wistar rats, showing that adequate Se intake markedly alleviates aortic endothelial damage observed under Se-deficient conditions. In contrast, Se deficiency was associated with crater-like surface defects, EC necrosis, platelet adhesion, and VSMC migration toward the intima, along with reduced GPx activity and an altered prostacyclin/thromboxane balance [156].

In addition to in vitro and animal evidence, human data also support this association. A cross-sectional study including 191 adults reported an inverse correlation between serum Se levels and carotid intima–media thickness—a structural marker of endothelial dysfunction and subclinical atherosclerosis—as well as endothelial adhesion molecules (VCAM-1, ICAM-1, E-selectin), which mediate leukocyte recruitment to dysfunctional endothelium, further corroborating the link between selenium and atherogenic alterations [151].

## 4. Conclusions

Available evidence supports a multifaceted role for Se in preserving vascular integrity and counteracting key processes involved in atherosclerosis development. Se contributes to the control of oxidative stress and inflammation, two central drivers of vascular injury, while also modulating autophagy and cellular homeostasis. These effects collectively mitigate endothelial dysfunction, a pivotal early event in atherogenesis, and help delay the progression of vascular aging. In addition to these well-established mechanisms, emerging evidence points to a role for Se in maintaining genomic stability. Se-dependent enzymes and nuclear selenoproteins can limit oxidative DNA damage, reduce chromosomal instability, and support telomere integrity. Given that DNA damage promotes cellular senescence and amplifies pro-inflammatory signaling, this pathway represents a promising but still underexplored component of Se’s vasculoprotective effects.

It is important to emphasize that Se should not be conceptualized as a simple or isolated antioxidant, but rather as a pleiotropic regulator of redox homeostasis acting through multiple interconnected and hierarchical mechanisms. Its antioxidant properties are primarily mediated by a network of selenoproteins, including GPx, TrxR, and SELENOP, which collectively regulate intracellular and extracellular ROS, lipid peroxidation, and redox-sensitive signaling pathways. However, the biological activity of Se extends beyond classical ROS detoxification and includes the modulation of endothelial function, inflammatory signaling cascades (such as NF-κB and MAPK pathways), mitochondrial integrity, and cellular stress responses, including autophagy and apoptosis.

Importantly, emerging evidence also supports a role for Se in processes that indirectly intersect with redox biology, such as the maintenance of genomic stability and the attenuation of oxidative DNA damage, both of which are tightly linked to the regulation of cellular senescence. In vascular cells, these mechanisms may converge to reduce endothelial dysfunction, delay vascular aging, and limit the progression of atherosclerotic lesions. Therefore, the antioxidant effects of Se should be interpreted within a broader systems biology framework, in which redox regulation is integrated with inflammatory, metabolic, and cell fate pathways rather than considered in isolation.

This multifaceted nature may also help explain the apparent discrepancies observed in clinical studies, where Se supplementation shows benefits predominantly in populations with low baseline Se status, while offering limited or even potentially adverse effects in selenium-replete conditions.

However, both deficiency and excess can disrupt redox balance, enhance oxidative stress, and impair endothelial function. Low Se status is consistently associated with higher cardiovascular risk, particularly for CVD incidence and CVD mortality, as shown by multiple observational meta-analyses. However, Se appears to be protective only within an optimal physiological range, while very high circulating levels or excessive intake may attenuate benefits or increase risk, supporting a nonlinear dose–response relationship. This U-shaped association likely contributes to the heterogeneity observed across clinical studies and underscores the importance of maintaining optimal, rather than supra-physiological, Se levels.

Despite significant mechanistic and preclinical support, translation into clinical practice remains challenging. RCTs do not provide strong evidence of reduced CHD/CVD events or mortality with Se supplementation alone, despite suggestive trends in some analyses. Nevertheless, evidence from observational studies and targeted interventions suggests that benefits may be more evident in individuals with low baseline Se status, whereas supplementation in Se-replete populations may confer little advantage or potential adverse effects. Evidence also suggests that Se-related benefits may depend on synergistic interactions with other antioxidants (e.g., vitamins A/C/E, zinc, β-carotene, coenzyme Q10), since mortality reductions have been observed mainly in combined supplementation strategies.

These findings highlight the need for a personalized approach to Se in cardiovascular prevention, taking into account baseline nutritional status, individual risk profiles, and possibly genetic variability in selenoprotein function. The potential toxicity threshold and upper safe range for cardiovascular outcomes remains poorly defined, warranting further investigation of high-normal and excessive Se exposure. Future research should prioritize well-designed, stratified clinical trials and mechanistic studies to better define dose–response relationships and identify reliable biomarkers of Se activity beyond circulating levels. In particular, further investigation into Se’s role in DNA damage and repair pathways in vascular cells may uncover novel therapeutic targets. The role of combined antioxidant regimens (Se plus co-antioxidants) should be evaluated in factorial trial designs to disentangle Se-specific effects from synergistic supplementation effects. Furthermore, dietary Se has been shown to modulate gut microbiota composition, which in turn influences the host’s Se status and the expression of selenoproteome [157]. In parallel, dysbiosis has been closely linked to the onset and progression of CVD through interconnected pathways involving immune–metabolic regulation, altered lipid metabolism, and endothelial dysfunction [158]. Consistently, altered levels of Selenomonadales and selenocompounds have been reported in patients with MI, with a marked reduction in Selenomonadales abundance in MI groups, providing initial evidence of a link between Se status, microbiota composition, and CVD and suggesting potential future preventive strategies in cardiovascular health [159].

Overall, Se emerges as a promising yet complex modulator of vascular function. A balanced, evidence-based approach will be essential to harness its protective effects while minimizing risks, ultimately contributing to more effective strategies for the prevention and management of atherosclerotic CVD.

## Figures and Tables

**Table 1:** Human studies investigating the association between selenium status/supplementation and cardiovascular outcomes.

Observational Studies							
**Reference**	**Study Design/** **Population Size**	**Mean Age/Sex**	**Follow-Up (Loss to Follow-Up if Reported)**	**Selenium Exposure Assessed**	**Cardiovascular** **Outcomes**	**Main Effect Estimates**	**Sex-Specific** **Findings**
Flores-Mateo et al. (2006) [115]	Meta-analysis of 14 prospective cohort + 11 case–control studies; sample size not specified	20–84 years; 0–59% female	3–25 years	Blood or toenail Se concentration	CHD incidence/mortality	Cohort studies: RR = 0.85 (95% CI: 0.74–0.99) comparing highest vs. lowest Se category; case–control studies: RR = 0.43 (95% CI: 0.29–0.66); 24% CHD risk reduction per 50% increase in Se concentration	Not reported
Zhang et al. (2016) [116]	Meta-analysis of 16 prospective studies; n = 35,607 participants; 4421 incident CVD cases	20–90 years; sex ratio not specified	3–15 years; 0–44% females	Blood Se concentration	Total CVD incidence	Significant inverse association within blood Se range 55–145 µg/L; association became null above 145 µg/L	Not reported
Xiang et al. (2020) [123]	Meta-analysis of 10 cohort and 2 case–control studies; 25,667 participants	20–85.8 years; 0–100% female	5–25.7 years	Circulating Se levels	All-cause mortality, CVD mortality, CHD mortality	Lowest vs. highest Se category: all-cause mortality RR = 1.36 (95% CI: 1.18–1.58); CVD mortality RR = 1.35 (95% CI: 1.13–1.62); CHD mortality RR = 1.43 (95% CI: 0.93–2.19; not significant)	Not reported
Kuria et al. (2021) [114]	Meta-analysis of observational studies and RCTs; 13 studies overall	Not reported	Not reported	Blood Se concentration	CVD incidence and CVD mortality	15% reduction in CVD incidence per 10 μg/L increase in blood Se; nonlinear dose–response association; lowest risk at 30–35 μg/L; risk increased above 300 μg/L	Not reported
Yang et al. (2022) [125]	Meta-analysis of 49 studies and 61 population cohorts; 1440 subjects with MI, 1917 subjects with CHD	Not reported	Not applicable	Blood/hair/urinary Se levels	MI and CHD prevalence	Lower Se levels in MI patients vs. controls: SMD = −3.64 (95% CI: −4.43 to −2.85); lower Se levels in CHD patients: SMD = −0.47 (95% CI: −0.67 to −0.28)	Not reported
Guo et al. (2024) [126]	Cross-sectional analysis NHANES 2011–2016; 5101 participants	65 years; 41.3% female	Not applicable	Blood Se concentration	CHD prevalence and CVD morbidity	Second quartile OR = 0.71 (95% CI: 0.53–0.96); third quartile OR = 0.73 (95% CI: 0.55–0.99); fourth quartile OR = 0.74 (95% CI: 0.55–0.98) vs. lowest quartile	Not reported
Cui et al. (2025) [127]	Meta-analysis of 20 population-based studies; 41,548 participants from 9 studies assessed for CVD mortality	37–87 years; sex ratio not specified	4.3–17.3 years	Serum Se and SELENOP concentration	CVD mortality	Each SD increase in Se/SELENOP associated with reduced CVD mortality: RR = 0.89 (95% CI: 0.84–0.94)	Not reported
Liang et al. (2025) [17]	Cross-sectional analysis NHANES 2003–2018; 39.372 participants	47.3 years; 51.8% female	Not applicable	Dietary Se intake	CVD, CHD, ASCVD prevalence	Highest vs. lowest tertile: CVD OR = 0.73 (95% CI: 0.63–0.86); CHD OR = 0.78 (95% CI: 0.64–0.95); ASCVD OR = 0.85 (95% CI: 0.74–0.98)	Stronger inverse association in females
Randomized controlled trials							
**Reference**	**Study design/** **population size**	**Mean age/sex**	**Follow-up duration**	**Selenium intervention**	**Cardiovascular** **outcomes**	**Main results**	**Sex-specific** **findings**
Flores-Mateo et al. (2006) [115]	Meta-analysis of 6 RCTs; 17,766 participants	47–62 years; 13–61% female	0.5–7.6 years	Se yeast/sodium selenite/not specified Se form alone or combined with vitamins/minerals (75–200 μg/day)	CHD incidence	Pooled RR = 0.89 (95% CI: 0.68–1.17); not statistically significant	Not reported
Zhang et al. 2016 [116]	Meta-analysis of 6 RCTs; 37,572 participants	35–69 years; sex ratio not specified	0.5 months–12 years	Se yeast/bio-Se/not specified Se form alone or combined with vitamins/minerals (75–200 μg/day)	CVD incidence	No significant effect on CVD risk	Not reported
Ju et al. 2017 [118]	Meta-analysis of 16 RCTs; 43,998 participants	40–85 years; 0–82% female	Not reported	Se alone or in combination with vitamins/minerals (50–500 μg/day)	CVD mortality/CHD mortality/inflammatory markers	Non-significant reduction in CHD mortality: OR = 0.88 (95% CI: 0.76–1.02); lower CRP levels and increased GPx activity	Not reported
Jenkins et al. 2020 [121]	Meta-analysis of 43 RCTs; sample size not specified	Not reported	Mean 10 years	Se alone or in combination with antioxidant mixtures	All-cause mortality/CVD mortality	Reduced cardiovascular mortality only when Se combined with antioxidants: RR = 0.77 (95% CI: 0.62–0.97); reduced all-cause mortality: RR = 0.90 (95% CI: 0.82–0.98)	Not reported
An et al. 2022 [122]	Meta-analysis of 884 RCTs; 883,627 participants	Not reported	Not reported	Oral Se supplementation	CHD, MI, CVD mortality	CHD RR = 0.89 (95% CI: 0.64–1.23); MI RR = 0.87 (95% CI: 0.59–1.30); CVD mortality RR = 0.97 (95% CI: 0.83–1.14)	Not reported

Abbreviations: ASCVD: atherosclerotic cardiovascular disease; CHD: coronary heart disease; CRP: C-reactive protein; CVD: cardiovascular disease; MI: myocardial infarction; GPx: glutathione peroxidase; NHANES: National Health and Nutrition Examination Survey; OR: odds ratio; RCT: randomized controlled trial; RR: relative risk; Se: selenium; SELENOP: selenoprotein P; SMD: standard mean difference.

## Data Availability

Data sharing is not applicable. No new data were created or analyzed in this study.

## Abbreviations

The following abbreviations are used in this manuscript:

Akt	Protein kinase B
AngII	Angiotensin II
ApoE^−/−^	Apolipoprotein E-deficient
ASCVD	Atherosclerotic cardiovascular disease
CHD	Coronary heart disease
CI	Confidence interval
Cuf-Se	Selenium-doped copper formate
CVD	Cardiovascular disease
DCs	Dendritic cells
DDR	DNA damage response
ECs	Endothelial cells
ECM	Extracellular matrix
eNOS	Endothelial nitric oxide synthase
GPx	Glutathione peroxidase
GSH	Glutathione
HUVECs	Human umbilical vein endothelial cells
ICAM-1	Intercellular adhesion molecule 1
IL	Interleukin
LDL	Low-density lipoprotein
MAPK	Mitogen-activated protein kinase
MCP-1	Monocyte chemoattractant protein 1
MDA	Malondialdehyde
MI	Myocardial infarction
MMP	Matrix metalloproteinase
mTOR	Mammalian target of rapamycin
NAD^+^	Nicotinamide adenine dinucleotide
NADH	Reduced nicotinamide adenine dinucleotide
NF-κB	Nuclear factor kappa-light-chain-enhancer of activated B cells
NLRP3	NOD-like receptor protein 3
NO	Nitric oxide
Nrf2	Nuclear factor erythroid 2-related factor 2
oxLDL	Oxidized low-density lipoprotein
OXPHOS	Oxidative phosphorylation
PGI_2_	Prostacyclin
PI3K	Phosphoinositide 3-kinase
PGE_2_	Prostaglandin E2
PPARγ	Peroxisome proliferator-activated receptor gamma
RCTs	Randomized controlled trials
ROS	Reactive oxygen species
SA-β-gal	Senescence-associated beta-galactosidase
SASP	Senescence-associated secretory phenotype
Se	Selenium
SeAlb	Selenium-containing albumin
SeCys	Selenocysteine
SELENOP	Selenoprotein P
SeMet	Selenomethionine
SeNPs	Selenium nanoparticles
SIRT	Sirtuin
SMD	Standard mean difference
SOD	Superoxide dismutase
TGF-β	Transforming growth factor beta
TNF-α	Tumor necrosis factor alpha
TrxR	Thioredoxin reductase
VCAM-1	Vascular cell adhesion molecule 1
VEGF	Vascular endothelial growth factor
VSMCs	Vascular smooth muscle cells
